# Iron-ing Out the Role of Transferrin Receptor in Kidney Development

**DOI:** 10.34067/KID.0000001193

**Published:** 2026-05-28

**Authors:** Leslie S. Gewin, Michael F. Romero

**Affiliations:** 1 Division of Nephrology, Department of Medicine, Washington University in St. Louis, St. Louis, Missouri; 2 Department of Medicine, Veterans Affairs Hospital, St. Louis VA, St. Louis, Missouri; 3 Physiology and Biomedical Engineering, Mayo Clinic College of Medicine and Science, Rochester, Minnesota; 4 Nephrology and Hypertension, Mayo Clinic College of Medicine and Science, Rochester, Minnesota; 5 Mayo Clinic Pirnie Polycystic Kidney Disease Center, Mayo Clinic College of Medicine and Science, Rochester, Minnesota

**Keywords:** cystic kidney, kidney development, mineral metabolism, polycystic kidney disease, transgenic mouse, nephron development

Nutritional deficiencies affect the developing fetus with both immediate effects (*e.g*., preterm deliveries, low birth weight) and by potentially increasing susceptibility to chronic illnesses such as hypertension and CKD which may develop decades later.^1^ Maternal iron deficiency, the most common deficiency, has been shown to affect organ development including the kidney. In preclinical models, maternal iron deficiency impairs nephrogenesis, potentially increasing the risk for hypertension and CKD later in life.^2^ Transferrin (Tf) is the primary carrier for iron, and Tf is delivered to tissues through binding to its receptor, transferrin receptor 1 (TfR1).^3^ While iron is important throughout gestation, the embryo only produces Tf at midgestation. Thus, the role of TfR1 in embryonic and perinatal kidney development remains unclear. In addition, several different lineages contribute to the developing kidney, and the effect of iron delivery to each lineage on kidney development is not known.

In the manuscript by Qiu *et al*.,^4^ many of these questions related to iron delivery and the developing kidney are addressed using elegant genetic studies to delete TfR1 in different lineages (mesenchymal, ureteric, and stromal) and comparing them with a model of global iron deficiency. The structures of the nephron are derived from either the ureteric bud (*e.g*., collecting duct and urinary system) or the metanephric mesenchyme (nephron proper, *i.e*., glomeruli and remaining tubular structures), and both express TfR1 at embryonic day E12.5–15.5. However, deletion of TfR1 in the ureteric bud (*Hoxb7Cre*) or metanephric mesenchyme (*Six2CreEGFP*) did not prevent ureteric bud or proximal tubule development (Figure 1, G and H versus Figure 1, A and B), respectively. TfR1 was not expressed in the stromal compartment, and not surprisingly, its deletion (*FoxD1Cre*) did not result in development abnormalities. Mice lacking TfR1 in the metanephric mesenchyme by *Six2Cre* (TfD) were not normal because there was a 50% reduction of glomeruli and proximal tubules and mild dysplasia. Even so, the phenotypes of these conditional knockout mice were milder than expected given the known importance of iron on the developing kidney. This was also illustrated by the authors' global iron deficiency model (FeD; Figure 1, D–F), generated by placing the breeder mice on an iron deficient diet before mating and throughout gestation. The FeD model led to increased fetal loss (44%) at midgestation, 75% loss of the litter by birth, hypoplasia with severe growth restriction across both ureteric bud-derived and mesenchymal-derived tubules (Figure 1, E and F).

**Figure 1:**
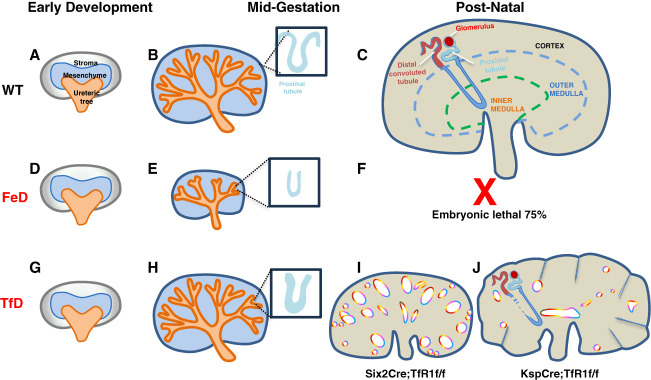
**Summary Model of Qiu *et al*, 2026 highlights.** A–C illustrate wild-type kidney development (metanephric kidney): (A) early kidney development, (B) midgestation, kidney development, and (C) postnatal and adult metanephric kidney. D–F are the same periods in mice on FeD illustrating that most FeD mice die before birth. G–J show the same time points for TfD. *Six2Cre*; *TfR(f/f)* removes TfR from metanephric mesenchymal stem cells of the metanephric kidney causing short and fewer proximal tubules, fewer cortical and medullary distal nephrons (*i.e*., uromodulin^+^ tubules), as well as metanephric cortical kidney cysts and reduced kidney function, increased serum creatinine (I). *KspCre:TfR(f/f)* removes TfR from distal nephrons (*i.e*., uromodulin^+^ tubules) in adult metanephric kidneys. This Ksp-TfR removal reduces HCT, increases serum creatinine, and increases BUN indicating more dramatically reduced kidney function. (Supplemental Figure 15 of ref. ^4^). FeD, iron-deficient diet; TfD, transferrin receptor deficient mice; TfR1, transferrin receptor 1.

The more severely impaired kidney development in the FeD model compared with the milder phenotypes of lineage-specific deletion of TfR1 cannot be entirely explained by incomplete recombination. The cells that escaped recombination (TfR1) did have a growth advantage and increased in number by birth. However, the authors also generated whole-kidney TfR1 knockout and did not show any gross development abnormalities through E11 (embryonic lethal after E12). These data suggest that while iron is critical for kidney development, the presence of TfR1 may not be required for nephrogenesis. So how does iron, vitally important to the embryo, reach the developing kidney? There are many potential avenues for iron to be delivered to tissues independent of TfR1 and collectively known as non–transferrin-bound iron (NTBI). Possible routes include ferritin internalization through the receptor Scara5^5^ and uptake of iron (Fe^2+^) through divalent metal transporter 1 (DMT1, NRAMP2; SLC11A2),^6^ zinc regulated transporter/iron regulated transporter-like protein 8/14 (ZIP8, SLC39A8; Zip14, SLC39A14),^6,7^ and transient receptor potential cation channel subfamily C member 6.^7^

While TfR1 has a limited role in embryonic development, this receptor was critical for perinatal kidney development. In mice with TfR1 knocked out in metanephric mesenchyme (*TfR1^Six2Cre^*; Figure 1I), significant cysts disrupted kidney morphology during the second postnatal week, and this progressed to almost 100% mortality by postnatal day 15. This phenotype seemed to be cell autonomous because mice with a distal tubule TfR1 deletion (*TfR1^KspCre^*) had preserved proximal tubules even at P60 (Figure 1J). Exactly how iron depletion leads to cystic/dysplastic kidneys is likely multifactorial because iron plays important roles that extend beyond its important role in hemoglobin-oxygen transfer. Both the genetic and iron-deficient diet models were characterized by impaired tubule proliferation, consistent with known iron promotion of cell cycle and proliferation. In addition to proliferative defects, there was extensive apoptosis in the *TfR1^Six2Cre^* kidneys. Iron also plays a critical role in mitochondrial function,^8,9^ and proximal tubules are densely packed with mitochondria to support tubular transport functions. Thus, developing proximal tubules may be particularly sensitive to iron deficiency. Interestingly, the cystic areas of the kidney had longer cilia, and many cilia-associated genes (*e.g*., *Nphp*) were suppressed in the TfR1 conditional knockout kidneys. In addition, kidneys with reduced expression of Wnt9b had decreased TfR1 expression and a similar phenotype of cystogenesis compared with the TfR1 genetic knockout mice. These findings are intriguing. Thus, future research should define whether (*1*) Wnt9b plays an upstream role in TfR1 expression and (*2*) the role of ciliary disruption in iron deficiency-induced cystogenesis.

The postnatal hypoplastic and cystic phenotype of *TfR1^Six2Cre^* kidneys (Figure 1I) was rescued when iron (ferric sucrose, Fe^3+^) was given at P8. These findings show that reduced iron is the mediator of abnormal postnatal development on deletion of TfR1. The response to iron might be somewhat surprising given that absence of TfR1 should abrogate perinatal delivery of Tf-bound iron. However, it may be that NTBI can compensate if iron levels are sufficient or that proliferation of the TfR1+ rogue cells enables most of the developing kidney to respond. Prior data have shown that hypoxia inducible factor activity is increased in both iron deficiency and cystic growth,^10^ making this a plausible link between the two. The authors treated *TfR1^Six2Cre^* mice with roxadustat, a prolyl-hydroxylase inhibitor which stabilizes hypoxia inducible factor leading to greater activity. Surprisingly, roxadustat improved the cystic phenotype. Roxadustat treatment augmented gene expression of kidney-derived *Epo*, encoding erythropoietin, and reduced liver-derived *Hamp* (hepcidin), which would potentially augment iron delivery to the kidney. The authors reasonably postulated that this increased iron delivery led to the improved postnatal kidney development.

What are the implications for human kidney development and the common problem of iron deficiency? These studies confirm the devastating consequences of dietary iron deficiency on the developing kidney. NTBI likely plays a more prominent role in early kidney development, but the Tf receptor was critical for iron delivery later in development. Could treatment with iron or prolyl-hydroxylase inhibitors be protective if given to babies of iron-deficient mothers at birth? Unfortunately, this may not be as effective because murine kidney development continues postnatally for a few weeks whereas human kidney development ends at birth (though may be extended postnatally in preterm infants). Future research could help define the window of opportunity to treat iron deficiency in human pregnancies. While iron supplementation starting early in pregnancy is ideal, the possibility of rescue therapy for those who come to medical attention later in pregnancy is attractive. The potential long-term consequences of iron deficiency on the developing kidney (*e.g*., hypertension, CKD) and other organs make this research incredibly important.

## Supplementary Material
