# Supplementary material for: Iron-ing Out the Role of Transferrin Receptor in Kidney Development
Source: Kidney360. 2026 May 28;7(5):932–4. doi: 10.34067/KID.0000001193 (PMC13229427; doi:10.34067/KID.0000001193)
Supplement: Supplementary file 1 [file kidney360-7-0932-s001.pdf]

ASN American Society of Nephrology | JASN Journal of the American Society of Nephrology | KIDNEY360 Accessing Our World From Every Angle | CJASN Clinical Journal of the American Society of Nephrology

ASN Journal Disclosure Form

As per ASN journal policy, I have disclosed any financial relationships or commitments I have held in the past 36 months as included below. I have listed my Current Employer below to indicate there is a relationship requiring disclosure. If no relationship exists, my Current Employer is not listed.

L. Gewin reports the following:
Employer: Washington University in St. Louis School of Medicine, St. Louis VA Hospital; Consultancy: Novo Nordisk; Research Funding: NIH, VA; Longer Life Foundation; Advisory or Leadership Role: Associate Editor for Kidney360; and Other Interests or Relationships: member of American Society of Nephrology and American Heart Association and American Physiological Society and Southern Society for Clinical Investigation.

I understand that the information above will be published within the journal article, if accepted, and that failure to comply and/or to accurately and completely report the potential financial conflicts of interest could lead to the following: 1) Prior to publication, article rejection, or 2) Post-publication, sanctions ranging from, but not limited to, issuing a correction, reporting the inaccurate information to the authors' institution, banning authors from submitting work to ASN journals for varying lengths of time, and/or retraction of the published work.

Name: Leslie S. Gewin
Manuscript ID: K360-2026-000249
Manuscript Title: Iron-ing out the role of transferrin receptor in kidney development
Date of Completion: March 4, 2026
Disclosure Updated Date: March 4, 2026

ASN American Society of Nephrology · JASN Journal of the American Society of Nephrology · KIDNEY360 Accessing Our World From Every Angle · CJASN Clinical Journal of the American Society of Nephrology

ASN Journal Disclosure Form

As per ASN journal policy, I have disclosed any financial relationships or commitments I have held in the past 36 months as included below. I have listed my Current Employer below to indicate there is a relationship requiring disclosure. If no relationship exists, my Current Employer is not listed.

M. Romero reports the following:
Employer: Mayo Clinic College of Medicine & Science; Patents or Royalties: Inventor of Patent 24.04.2024 = European Patent Specification EP f3 740 122 B1: Tracer agent for use in an in vivo diagnostic method for diagnosing a patient's renal function; and Advisory or Leadership Role: Kidney360, Associate Editor; ASN Continuous Professional Development Committee [1/1/24-12/31/25]; NIDDK study sections (ad hoc); AHA study sections (ad hoc).

I understand that the information above will be published within the journal article, if accepted, and that failure to comply and/or to accurately and completely report the potential financial conflicts of interest could lead to the following: 1) Prior to publication, article rejection, or 2) Post-publication, sanctions ranging from, but not limited to, issuing a correction, reporting the inaccurate information to the authors' institution, banning authors from submitting work to ASN journals for varying lengths of time, and/or retraction of the published work.

Name: Michael F. Romero
Manuscript ID: K360-2026-00024
Manuscript Title: Iron-ing out the role of transferrin receptor in kidney development
Date of Completion: March 4, 2026
Disclosure Updated Date: December 19, 2025
